# Cost-Effectiveness of Thrombolysis within 4.5 Hours of Acute Ischemic Stroke in China

**DOI:** 10.1371/journal.pone.0110525

**Published:** 2014-10-20

**Authors:** Yuesong Pan, Qidong Chen, Xingquan Zhao, Xiaoling Liao, Chunjuan Wang, Wanliang Du, Gaifen Liu, Liping Liu, Chunxue Wang, Yilong Wang, Yongjun Wang, for the TIMS-CHINA investigators

**Affiliations:** 1 Department of Neurology, Beijing Tiantan Hospital, Capital Medical University, Beijing, China; Groningen Research Institute of Pharmacy, Netherlands

## Abstract

**Background:**

Previous economic studies conducted in developed countries showed intravenous tissue-type plasminogen activator (tPA) is cost-effective for acute ischemic stroke. The present study aimed to determine the cost-effectiveness of tPA treatment in China, the largest developing country.

**Methods:**

A combination of decision tree and Markov model was developed to determine the cost-effectiveness of tPA treatment versus non-tPA treatment within 4.5 hours after stroke onset. Outcomes and costs data were derived from the database of Thrombolysis Implementation and Monitor of acute ischemic Stroke in China (TIMS-China) study. Efficacy data were derived from a pooled analysis of ECASS, ATLANTIS, NINDS, and EPITHET trials. Costs and quality-adjusted life-years (QALYs) were compared in both short term (2 years) and long term (30 years). One-way and probabilistic sensitivity analyses were performed to test the robustness of the results.

**Results:**

Comparing to non-tPA treatment, tPA treatment within 4.5 hours led to a short-term gain of 0.101 QALYs at an additional cost of CNY 9,520 (US$ 1,460), yielding an incremental cost-effectiveness ratio (ICER) of CNY 94,300 (US$ 14,500) per QALY gained in 2 years; and to a long-term gain of 0.422 QALYs at an additional cost of CNY 6,530 (US$ 1,000), yielding an ICER of CNY 15,500 (US$ 2,380) per QALY gained in 30 years. Probabilistic sensitivity analysis showed that tPA treatment is cost-effective in 98.7% of the simulations at a willingness-to-pay threshold of CNY 105,000 (US$ 16,200) per QALY.

**Conclusions:**

Intravenous tPA treatment within 4.5 hours is highly cost-effective for acute ischemic strokes in China.

## Introduction

Stroke accounts for 301 million disability-adjusted life-years, making it the first leading cause of death and imposing significant disease and economic burden in China [1]. Randomized controlled trials and large observational studies have demonstrated the effectiveness with acceptable safety of intravenous recombinant tissue-type plasminogen activator (tPA) for patients within 4.5 hours after onset of acute ischemic stroke [2]–[6].

Economic studies conducted in North America, Europe and Australia showed tPA given within 4.5 hours is cost-effective or even cost-saving in the long term [7]–[15]. However, all of these studies were conducted in developed countries; the findings from these countries may not be relevant in developing countries due to their differences in demographics, healthcare systems and payment coverage. Compared with developed countries, the low- and middle- income countries suffer higher mortality burden of stroke [16], and have lower percentage of patients with ischemic stroke treated with tPA [17]. On the other hand, most developed countries, such as United States, implement prospective payment system based on diagnosis related groups [18], [19]. While in China, the payments are based on each clinical service. Particularly, the drug costs account for a large part of total costs for Chinese stroke patients. They pay CNY 8,197 (US$ 1,261) just for 70 mg tPA alone [20]. Is the expensive tPA still cost-effective and should it be widely generalized in developing countries like China? Economic analysis of tPA in developing countries is urgent.

Little is known about verifying the cost-effectiveness of tPA treatment in China. We sought to determine the cost-effectiveness of tPA within 4.5 hours after onset of acute ischemic stroke, using the data from the Thrombolysis Implementation and Monitor of acute ischemic Stroke in China (TIMS-China), which is a nationwide prospective registry of thrombolytic therapy with intravenous tPA in patients with acute ischemic stroke between May 2007 and April 2012. TIMS-China has recruited 1,440 consecutive tPA treated patients from 67 centers [21].

## Methods

### Model Overview

**Figure 1:**
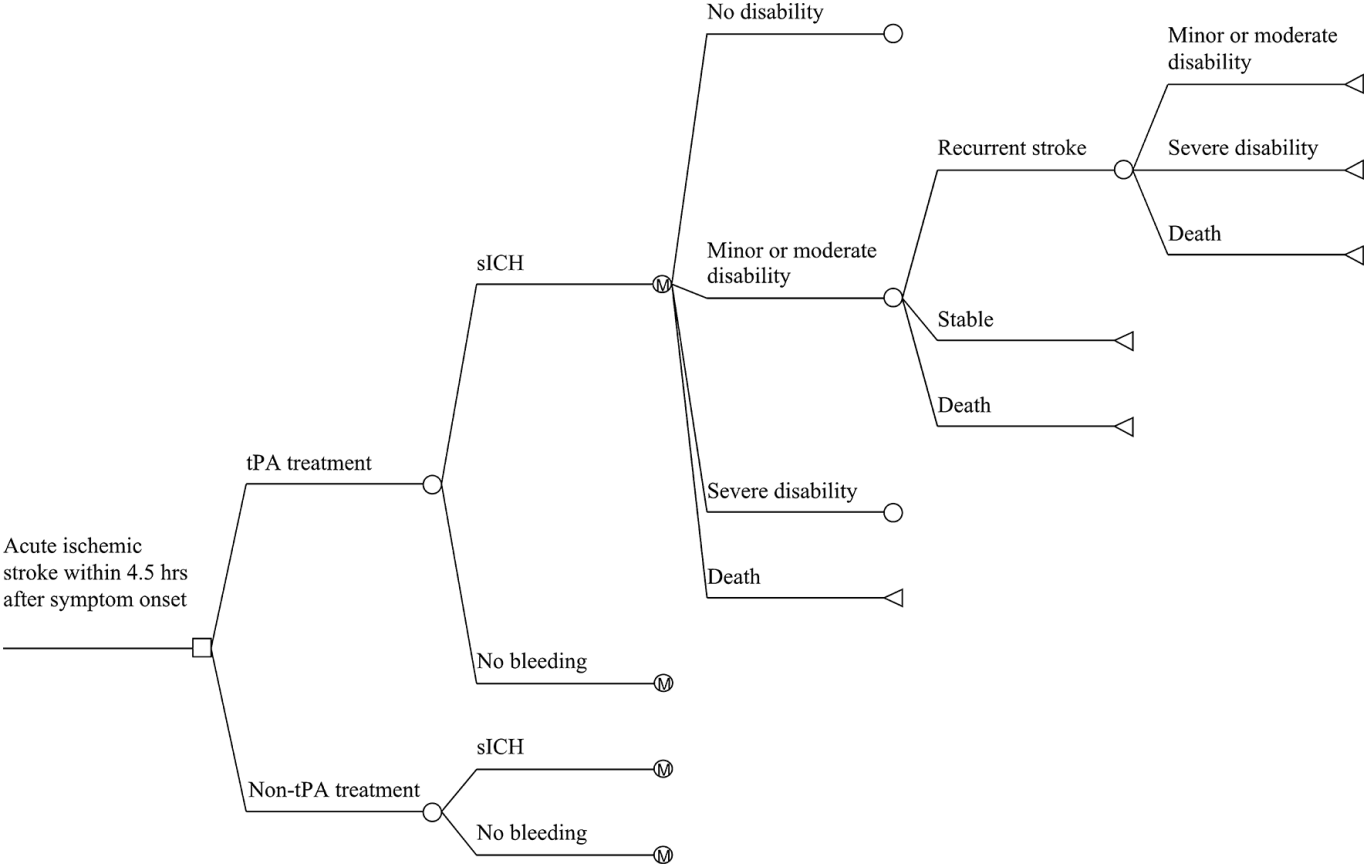
Markov state-transition model. All modeled patients started at 63 years old with an acute ischemic stroke receiving either intravenous tissue-type plasminogen activator (tPA) or no tPA treatment within 4.5 hours after the onset of stroke, and transition between health states was tracked until death or the 30-year time horizon is reached. Patients remaining alive after recurrent stroke may transit to category of equal or greater disability. Only transition from minor or moderate disability category was illustrated in the figure. M = Markov node; sICH =  symptomatic intracerebral hemorrhage.

We adhered to the recommendations of the Panel on Cost-effectiveness in Health and Medicine [22], including (1) components belonging in the numerator and denominator of a cost-effectiveness (C/E) ratio; (2) measuring terms in the numerator of a C/E ratio; (3) valuing health consequences in the denominator of a C/E ratio; (4) estimating effectiveness of interventions; (5) incorporating time preference and discounting; and (6) handling uncertainty. A combination of decision tree and Markov model (Figure 1) was developed to simulate the long-term (30 years) cost-effectiveness of tPA treatment versus absence of tPA treatment within 4.5 hours after the onset of stroke. Our study was based on data from 1128 patients with acute ischemic stroke who received intravenous tPA within 4.5 hours in TIMS-CHINA [21]. The base case of model was a cohort of 100,000 patients (39% female), with mean age of 63 years old, arriving at hospital within 4.5 hours after onset of stroke, whose clinical and demographic characteristics are same as patients enrolled in TIMS-CHINA study (Table 1). Total costs and quality-adjusted life-years (QALYs) gained with each alternative were estimated for each health state at 90 days from the index events and then estimated annually for the remainder 30 years. This analysis was conducted from the perspective of healthcare payers, including the government, medical insurance and patients.

**Table 1:** Clinical and Demographic Characteristics of Patients Treated with Tissue Plasminogen Activator within 4.5 Hours in TIMS-CHINA Study (N = 1128).

Characteristic	
Age (year), mean (SD)	63.48(11.34)
Female, n(%)	440(39.01)
Preadmission modified Rankin Score 0–1, n(%)	1081(95.83)
NIHSS at admission, median(Q1–Q3)	11(7–16)
Hypertension, n(%)	667(59.13)
Diabetes mellitus, n(%)	196(17.38)
Dyslipidemia, n(%)	73(6.47)
Atrial fibrillation, n(%)	202(17.91)
Current smoking, n(%)	387(34.31)
History of stroke, n(%)	208(18.44)

TIMS-CHINA, Thrombolysis Implementation and Monitor of acute ischemic Stroke in China.

### Input Parameters

The baselines of patients and their outcomes in the three time windows (0–1.5, 1.5–3, 3–4.5 hours after the onset of stroke) of the tPA group, were obtained from the observed data directly drawn from the TIMS-CHINA study database (Table 2). Odds ratios of the favorable functional outcome (modified Rankin Scale (mRS) 0–1), death and symptomatic intracerebral hemorrhage (sICH) in each time window were derived from a pooled analysis of the European Cooperative Acute Stroke Study Trial (ECASS), the Alteplase Thrombolysis for Acute Noninterventional Therapy in Ischemic Stroke Trial (ATLANTIS), the National Institute of Neurological Disorders and Stroke (NINDS), and the Echoplanar Imaging Thrombolytic Evaluation Trial (EPITHET) [23]. Odds ratio of sICH within 0–1.5 hours were assumed to be the same as that of within 1.5–3 hours. Favorable functional outcome, death and sICH rates in the non-tPA treatment group were obtained from the observed data of the tPA group and the odds ratios of previous study [23]. The proportional distribution of patients remaining in categories of mRS 2–3 and mRS 4–5 at 90 days in the control group was assumed to be the same as that in tPA group.

**Table 2:** Outcomes and Efficacy of Patients Treated with tPA by Different Time From Onset to Needle.

Model Input	0–1.5 hrs	1.5–3 hrs	3–4.5 hrs	Reference
Percentage of patients	5.68	61.13	33.19	TIMS-CHINA database
Functional outcome at 90 days after tPA treatment (%):				
No disability (mRS 0–1)	53.97	47.11	48.09	
Minor or moderate disability (mRS 2–3)	23.81	24.89	25.14	
Severe disability (mRS 4–5)	9.52	18.37	15.30	
Death (mRS 6)	12.70	9.63	11.47	
sICH (per ECASS II definition)	4.69	3.05	5.35	
Odds ratio (95% CI) at day 90				
mRS 0–1	2.55	1.64	1.34	23
	(1.44–4.52)	(1.12–2.40)	(1.06–1.68)	
Death	0.78	1.13	1.22	23
	(0.41–1.48)	(0.70–1.82)	(0.87–1.71)	
sICH	8.23	8.23	3.61	23
	(2.39–28.32)	(2.39–28.32)	(1.76–7.38)	

tPA, tissue plasminogen activator; mRS, modified Rankin Score; sICH, symptomatic intracerebral hemorrhage; ECASS II, Second European-Australasian Acute Stroke Study; TIMS-CHINA, Thrombolysis Implementation and Monitor of acute ischemic Stroke in China.

Recurrent rates of stroke and mortality rates of recurrent strokes in years after the first 90 days were estimated from the China National Stroke Registry (CNSR, a nationwide registry for patients with acute cerebrovascular events in China between September 2007 and August 2008, recruiting 21,902 consecutive patients from 132 hospitals in China) [24]. We further assumed an increase in stroke recurrence rates by 1.019-fold per life year, according to the relative risk estimated from patients of ischemic stroke in CNSR.

Age specific non-stroke mortality rates were derived from the most recent published census of China and adjusted by the causes of death of 2010 reported in the China Health Statistics Yearbook 2012 [25], [26]. Disability status were assumed to affect survival rate, therefore the final age specific non-stroke death rates in the model were adjusted according to the mRS-specific death hazard ratios [7], [27]. Patients remaining alive after stroke recurrence were assumed to be reallocated equally among categories of equal and greater disability [12]. For example, patients with minor or moderate disability who had a recurrent stroke and survived were allocated equally among minor or moderate disability category and severe disability category.

### Costs

The total costs including both out-of-pocket costs and reimbursements, were converted to 2011 Chinese Yuan Renminbi (CNY) using the medical care component of consumer price index [26]. The average cost of single hospitalization after stroke was obtained from CNSR and the China Health Statistics Yearbook 2012 [26]. Annual post-hospitalization costs (such as inpatient and outpatient rehabilitation, ambulatory care and second prevention costs) were also obtained from CNSR. We estimated the additional costs of tPA treatment and occurrence of sICH after thrombolysis using the data from CNSR and TIMS-CHINA. Indirect economic costs such as lost work productivity were not included in this study. All costs and utilities were discounted by 3% per year [12].

### Health States

Patients could undergo transitions between four disability states according to functional outcome based on mRS: no disability (mRS 0–1), minor or moderate disability (mRS 2–3), severe disability (mRS 4–5) and dead (mRS 6) [28]. At the end of each cycle, patients could remain in their current health state, transit to a lower level health state due to recurrent stroke, or die due to a recurrent stroke or a non-stroke cause (see Figure 1).

### Outcome Assessment

Health outcomes were measured in QALYs by multiplying years of life by utility scores derived from the literature. Utility estimates were based on published utility values stratified by mRS category [12], [29], [30]. Economic outcomes were measured in cumulative direct medical costs associated with stroke. The incremental cost-effectiveness ratio (ICER) was calculated by dividing the incremental costs by the incremental QALYs. We modeled outcomes and costs over the short-term (2 years) and the long-term (30 years). Using the willingness-to-pay threshold recommended by the Commission on Macroeconomics and Health of World Health Organization [31], the intervention was considered cost-effective if the ICER was less than CNY 105,000 (3x GDP per capita of China in 2011 [26], US$ 16,200) per QALY gained.

### Sensitivity Analysis

The robustness of the model was tested by means of one-way sensitivity analyses for all variables across plausible ranges, which were obtained from the literature (Table 2, Table 3). To evaluate the impact of the parameter and stochastic uncertainty in all variables varied simultaneously, a probabilistic sensitivity analysis was performed using Monte Carlo simulation in Ersatz v1.3 (a bootstrap add-in for Microsoft Excel for Windows; EpiGear International Pty Ltd, Brisbane, Australia). We assumed the probabilities and utilities followed a beta distribution, and costs followed a lognormal distribution. The simulation was run 10,000 times to capture stability of the results. A scatter-plot and a cost-effectiveness acceptability curve were developed to represent uncertainty. Sensitivity analyses were only applied to the long-term (30 years) results.

**Table 3:** Base-case and the Range of Values Used in the Model.

Model Input	Base Case	Range	Reference
**Probabilities inputs**			
Recurrent rate of stroke (per patient year)	0.1181	0.1123–0.1241	CNSR
Relative risk of stroke recurrence per life year	1.019	1.014–1.024	CNSR
Death rate with recurrent stroke	0.2101	0.1887–0.2316	CNSR
Age specific non-stroke death rate*	0.0089–0.1654		25,26
Non-stroke death hazard ratios			27
mRS 0–1	1	1.0–1.2	
mRS 2–3	1.19	1.1–1.3	
mRS 4–5	2.04	1.4–3.0	
**Cost inputs (2011 Chinese Yuan Renminbi)**			
Additional costs of tPA treatment	10830	8385–12630	CNSR, TIMS-CHINA
Additional costs of sICH	2300	500–4800	TIMS-CHINA
One-time hospitalization costs			
mRS 0–1	9526	5502–11994	CNSR, 26
mRS 2–5	12595	6922–16516	
mRS 6	10794	5072–14267	
Annual post-hospitalization costs			
mRS 0–1	6773	2028–8639	CNSR
mRS 2–5	10305	2592–12959	
**Utility inputs**			
No disability (mRS 0–1)	0.80	0.80–0.95	12,29,30
Minor or moderate disability (mRS 2–3)	0.58	0.56–0.78	
Severe disability (mRS 4–5)	0.28	0.05–0.36	
Death (mRS 6)	0.00	0.00–0.00	
**Discount rate inputs**			
Costs	0.03	0.03–0.08	12
Outcomes	0.03	±20%	12

All costs were converted to 2011 Chinese Yuan Renminbi by using the medical care component of consumer price index; to convert CNY to US dollars, divide by 6.5.

tPA, tissue plasminogen activator; mRS, modified Rankin Score; sICH, symptomatic intracerebral hemorrhage; CNSR, China National Stroke Registry. TIMS-CHINA, Thrombolysis Implementation and Monitor of acute ischemic Stroke in China.

*Age specific non-stroke death rate: only the number of 63 years old (0.0089) and 93 years old (0.1654) are presented.

## Results

### Base Case Analysis

Table 4 shows the outcomes, costs and ICER calculated in the short term (1, 2 years) and in the long term (30 years). In the base-case scenario, for a 63-year-old patient with acute ischemic stroke, tPA treatment would be cost-ineffective in the first year, but become cost-effective from the second year onwards, using the threshold of CNY 105,000 (3x GDP per capita of China in 2011, US$ 16,200) as the willingness-to-pay per QALY. After 2 years, tPA treatment gained 0.101 QALYs at an additional cost of CNY 9,520 (US$ 1,460), yielding an ICER of CNY 94,300 (US$ 14,500) per QALY gained. In the long term (30 years), tPA treatment gained 0.422 QALYs at an additional cost of CNY 6,530 (US$ 1,000), yielding an ICER of CNY 15,500 (US$ 2,380) per QALY gained.

**Table 4:** Costs and Outcomes per Capita in Base-case Analysis.

Time horizon	Treat strategy	QALYs	Cost (CNY)	ICER (CNY/QALY)
1 year	No tPA	0.655	20,680	-
	tPA	0.711	30,850	181,607
2 years	No tPA	1.102	29,460	-
	tPA	1.203	38,980	94,257
30 years	No tPA	4.571	114,350	-
	tPA	4.993	120,880	15,474

tPA, tissue plasminogen activator; QALY, quality-adjusted life-year; ICER, incremental cost-effectiveness ratio.

### Sensitivity Analysis

**Figure 2:**
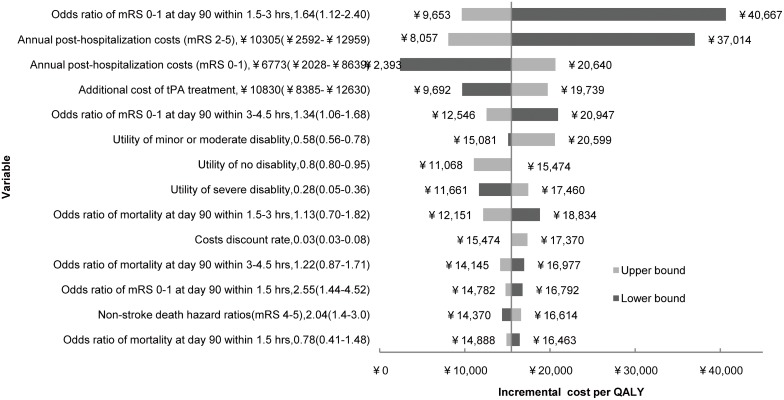
One-way sensitivity analyses on incremental cost-effectiveness ratio (ICER) gained in the long term (30 years) by tPA treatment within 4.5 hours. All model parameters were analyzed, and only those with the highest relative effect on ICER are displayed. Dark shaded bars represent the lower bound, while light shaded bars represent the upper bound of the parameter range. Base-case scenario of ICER is CNY 15,500 per quality-adjusted life-year (QALY) gained.

One-way sensitivity analysis showed the results in the long term are robust. Tornado diagrams illustrated the effect of varying input parameters on the long term ICER (Figure 2). Overall, the ICER was most sensitive to odds ratio of favorable functional outcome at day 90 within 1.5–3 hours and annual post-hospitalization costs (mRS 2–5). If odds ratio of favorable functional outcome at day 90 within 1.5–3 hours increased to 2.40 from 1.12, the ICER of tPA treatment would drop to CNY 9.653/QALY from CNY 40,667/QALY. If annual post-hospitalization cost of disabling stroke (mRS 2–5) increased from CNY 2,592 to CNY 12,959, the ICER would decrease from CNY 37,014/QALY to CNY 8,057/QALY. In contrast, the ICER was relatively insensitive to odds ratio of sICH at day 90 and one-time hospitalization costs.

**Figure 3:**
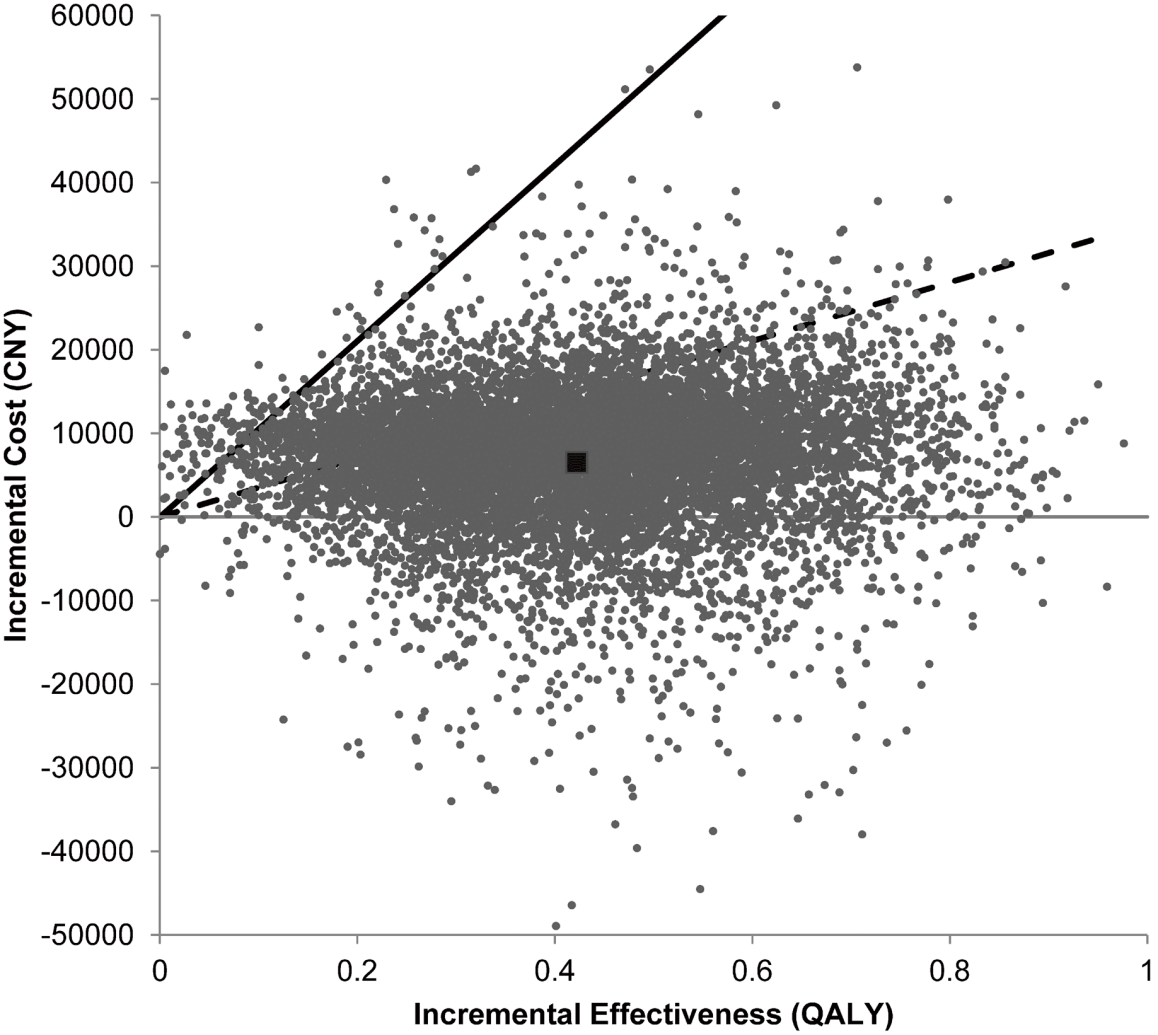
Scatterplot of the result of probabilistic sensitivity analysis comparing tPA treatment within 4.5 hours with no tPA treatment. Each point represents a simulation. The dark square represents the base case (0.422 QALYs gained at an incremental cost of CNY 6,530). The solid line represents the willingness-to-pay threshold of CNY 105,000 per QALY. The dashed line represents CNY 35,100 per QALY. Points to the right of the willingness-to-pay line are considered cost-effective.

**Figure 4:**
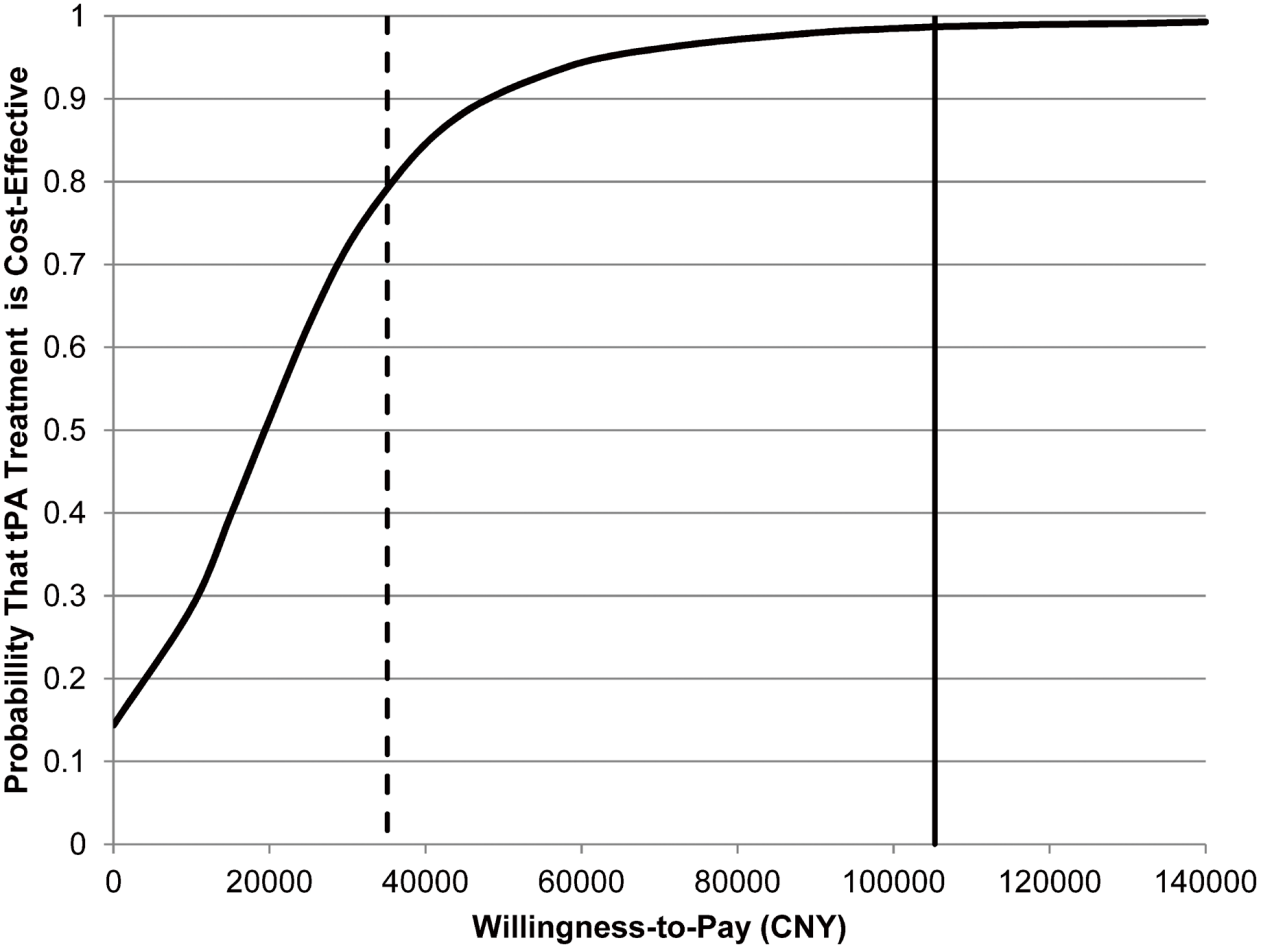
Cost-effectiveness acceptability curve. The curve presents the probability that tPA treatment within 4.5 hours to be cost-effective against willingness-to-pay threshold. The solid line represents the willingness-to-pay threshold of CNY 105,000 per QALY. The dashed line represents CNY 35,100 per QALY.

Results of the probabilistic sensitivity analysis in the long term are shown in Figure 3. Among the 10,000 simulation runs, there was a 14.4% chance that tPA treatment turned to be less costly and more effective than non-tPA treatment. tPA treatment was cost-effective in 98.7% of the simulations at a willingness-to-pay threshold of CNY 105,000 (3x GDP per capita of China in 2011, US$ 16,200) per QALY, and still remained cost-effective in 79.2% of the simulations at a threshold of CNY 35,100 (1x GDP per capita of China in 2011, US$ 5,400) per QALY. The cost-effectiveness acceptability curve of tPA treatment is shown in Figure 4.

## Discussion

Our study indicated that tPA treatment for acute ischemic stroke within 4.5 hours was cost-effective not only in the short term (2 years), but also highly cost-effective in the long term (30 years) in China. A patient with acute ischemic stroke treated with tPA within 4.5 hours gain an ICER of CNY 15,500 per QALY, which was below 1x GDP per capita of China in 2011 (CNY 105,000). By contrast, cost effectiveness analyses conducted in some developed countries demonstrated tPA treatment was cost-saving in the long term [7]–[10]. We also found that the ICER is less sensitive to the additional costs of tPA treatment, but more sensitive to odds ratio of favorable functional outcome at day 90 within 1.5–3 hours and annual post-hospitalization costs (Figure 2). The reason might be that tPA treatment is highly effective in functional outcome for acute ischemic stroke (OR = 1.64 for 1.5–3 hours [23]), and also dramatically influence the long-term utility and costs, which may offset the costs of tPA treatment.

The differences between China and the developed countries may influence the cost-effectiveness of tPA treatment. First, unlike the diagnosis related payment system in most developed countries [18], [19], China implements payment system according to the clinical services. The stroke-care related payments in China included medical care covered by government, medical insurance for urban workers, rural cooperative medical care, commercial medical insurance, plus patient own expense, in the proportions of 7.2%, 53.6%, 16.9%, 0.7% and 21.6%, respectively, according to CNSR (unpublished data). The out-of-pocket costs were only 10% to 20% of the total costs if the payment was covered by government. For urban workers with medical insurance, their average 3-month hospital and medication costs due to stroke were CNY 16,525 (US$ 2,361), while the out-of-pocket costs were CNY 14,478 (US$ 2,068) in 2006 [32]. However, for the patients without coverage from the government or medical insurance, they had to pay more out-of-pocket costs, with little support from state. No matter which payment resources that the stroke patients had, the total costs depended on the service and medicine prescribed, with huge variation in hospitals and geographical regions in China. In brief, Chinese patients must pay for all the costs first, and then search for corresponding reimbursement after the therapy process finished. For the patients without tPA treatment, their one-time hospitalization cost was CNY 9,526 if they had mRS 0–1, while if they had tPA treatment, the cost due to tPA alone was CNY 8,197 (US$ 1,261) in China [20]. Therefore, the cost of tPA in China were relatively higher than that in developed countries. Second, several surveys showed the lack of organized rehabilitation and decreased adherence to secondary prevention of ischemic stroke in China after discharge [33]–[36]. Therefore, the long-term post-hospitalization care costs might be similar between the tPA treatment group and the control group in China. Due to the facts above, clinically, it is very important to estimate the cost-effectiveness in China. And these facts might partly explain the difference in ICER between China and developed countries.

This study has several limitations. First, the costs used in the model may not be accurate for each component of tPA related treatments, tPA drugs, extra MRI scanning, extra consultant and nursing services, change in length of stay, and so on [10]. However, we think the overall costs were recorded accurately in the total hospitalization cost. We estimated the additional costs of tPA treatment on the basis of the difference of the total hospitalization costs of sICH-free patients with tPA treatment in TIMS-CHINA study and the hospitalization costs of patients without tPA treatment in CNSR. We calculated the additional costs of sICH applying the same rationale. Second, in China, the family plays an important role in both acute and post-acute care. This informal caregiving can be quite expensive. However, it was not considered in our model because of lack of corresponding data. Third, our model focused on the influence of tPA for acute ischemic stroke, and functional status and costs as a result of other causes, such as recurrence of intracranial hemorrhage, myocardial infarction, congestive heart failure, were not included in this model. In addition, the improved functional status after rehabilitation or other improvements was not considered in the model for lack of organized rehabilitation in China and lack of authentic data available on efficacy of rehabilitation or other improvements. Fourth, the efficacy of tPA treatment was based on the pooled analyses from the studies in developed countries (ECASS, ATLANTIS, NINDS, and EPITHET). Also, the utilities references were from Western literatures. We applied the efficacy of tPA treatment and the utilities to the model of Chinese patients, but did not know if these data fit Chinese patients. However, we ran sensitivity analysis and found that the results were robust for these parameters across plausible ranges. Although these limitations would have led to under- or over- estimation of the true cost-effectiveness of tPA treatment in China, it is unlikely to make a significant differential impact on the overall results of our study because our findings were robust as shown in the sensitivity analyses.

## Conclusions

The intravenous tPA within 4.5 hours for patients with acute ischemic stroke is highly cost-effective in China. Our study provides with constructive information on medical resource allocation for stroke treatment in China in future, and may also be a reference to other developing countries.
